# Synergistic neuroplasticity from synchronous Taiji Yunshou and tDCS in stroke: an fNIRS study of cortical activation and cross-subject hemodynamic brain network

**DOI:** 10.3389/fneur.2026.1818964

**Published:** 2026-05-28

**Authors:** Zhenguo Lin, Junwei Wang, Qi Xu, Wenhang Que, Taibiao Li, Zhenwu Zhang, Tiebin Yan

**Affiliations:** 1 Department of Clinical Medicine, Xiamen Medical College, Xiamen, China; 2 Department of Traditional Chinese Medicine, The Fifth Hospital of Xiamen, Xiamen, China; 3 Jiangxi Medical College, Nanchang University, Nanchang, China; 4 Department of Rehabilitation Medicine, Sun Yat-sen Memorial Hospital, Sun Yat-sen University, Guangzhou, China

**Keywords:** cross-subject hemodynamic brain network, functional near-infrared spectroscopy (fNIRS), stroke, Taiji Yunshou (TY), transcranial direct current stimulation (tDCS)

## Abstract

**Background:**

Stroke is a global, major and disabling non-communicable disease. Among acute stroke survivors, 50%−80% will experience upper limb dysfunction. While Taiji Yunshou (TY) and transcranial direct current stimulation (tDCS) demonstrate individual efficacy, the neurophysiological effects and mechanisms of their synchronous application at the hemodynamic brain network level remain unclear. This study compared the transient neurophysiological effects of the isolated vs. combined interventions on cortical activation and cross-subject hemodynamic brain network topology in left-hemispheric stroke.

**Methods:**

10 participants underwent three randomized 20-min brief interventions with 2-day washout periods: TY alone, tDCS alone, and synchronous TY + tDCS. Functional near-infrared spectroscopy (fNIRS) quantified the difference between post-intervention and pre-intervention oxygenated hemoglobin concentration changes (ΔHbO) under different conditions for intervention across left and right prefrontal cortex LPFC, RPFC, premotor and supplementary motor cortex LPMC, RPMC, and sensorimotor cortex LSMC, RSMC. The differences in ΔHbO among the three brief interventions were analyzed using Friedman test with Nemenyi test for *post hoc* pairwise comparisons. Cross-subject hemodynamic brain networks were also constructed using Graphical Least Absolute Shrinkage and Selection Operator (GLASSO) for three brief interventions, with community detection, nodal centrality metrics, edge betweenness, and global indicators also analyzed.

**Results:**

Synchronous TY + tDCS induced superior LPFC and RPFC activation vs. tDCS alone (LPFC: *p* < 0.01; RPFC: *p* < 0.05). tDCS alone and TY + tDCS both elicited greater LPMC activation than TY alone (*p* < 0.001). Each intervention caused specific network reorganization. TY formed motor-execution and cognitive-control communities. tDCS created a bipartite division with LSMC-RPMC inhibitory connection. TY + tDCS segregated LPFC and RPFC into a cognitive community in common with LPFC-RPMC and LPFC-LSMC inhibitory connections, LSMC as the principal hub and optimized global network efficiency.

**Conclusion:**

Synchronous TY + tDCS induced synergistic neuroplasticity through dual mechanisms, enhancing cognitive-motor integration and LSMC-driven interhemispheric network reorganization. These findings provide clinical neuroimaging evidence at the channel level and the cross-subject level for integrated rehabilitation paradigms and identify LSMC as a critical target for stroke recovery.

**Clinical trial registration:**

https://www.chictr.org.cn/, identifier ChiCTR2400088853.

## 1 Introduction

As the second leading cause of death and the third most common cause of disability among non-communicable diseases worldwide (1), stroke significantly limits patients' activities of daily living by impairing motor function and reducing mobility (2). Over 12.2 million new stroke cases occur globally each year. Approximately 50%−80% of acute stroke survivors develop upper limb impairment post-stroke, and around 50% of patients continue to experience upper limb impairment in the chronic phase, 6 months after onset (3). Therefore, exploring more effective rehabilitation strategies to promote neural function recovery has become a core challenge urgently needing resolution in contemporary rehabilitation medicine.

Tai Chi is one of the most renowned classical Chinese martial arts, which also provides health benefits to people. Apart from its positive impact on cardiopulmonary function and psychological status, multiple randomized controlled trials have confirmed that Tai Chi can be helpful in better adjusting physical control, obviously improving balance, enhancing limb stability, and improving gait symmetry in stroke patients, as well as effectively reducing fall risk (4–6). Taiji Yunshou (TY), a core movement of Tai Chi, is characterized by a three-dimensional dynamic coordination pattern through circular upper limb movements and simultaneous rotation of the waist and hips. TY can offer distinct advantages such as simplicity, ease of learning and memory, and high patient compliance (7).

Transcranial direct current stimulation (tDCS) is a safe, non-invasive neuromodulation therapy that regulates cortical excitability by applying weak direct current to the scalp (8). It is characterized by simplicity of operation, low cost, and good patient tolerance (9). Research has indicated the modulating effect of tDCS on various cortical functions, including emotion regulation, cognition, and memory (10–12). In stroke rehabilitation, the application of tDCS extends to improving executive function. Long-term follow-up data show that it can reduce limb spasticity and accelerate functional recovery (13, 14).

However, despite the proven efficacy of both TY and tDCS in stroke rehabilitation, the neural mechanisms and clinical synergistic effects of their combined use have not been systematically verified. The field of stroke neurorehabilitation is currently witnessing the emergence of multimodal combined intervention paradigms (15, 16). In this context, understanding the mechanisms of rehabilitation interventions from the angle of brain network is particularly important. Classic hemodynamic brain network analysis typically characterizes inter-regional real-time functional connectivity and dynamic information exchange by calculating time-series correlations within individual subjects. This brain network perspective is particularly crucial in post-stroke motor rehabilitation, where functional recovery fundamentally relies on the dynamic reorganization of cortical network synergy rather than the isolated activation of single regions (17, 18). However, traditional approaches that focus on transient states often compromise the robust quantification of such system-wide cortical synergy across the population. To overcome this, the cross-subject hemodynamic brain network introduced in this study extends the scope of analysis to collective phenotype interdependence, thereby filtering out inherent momentary fluctuation noise in time-series. To the best of our knowledge, no previous studies have applied cross-subject hemodynamic brain networks to investigate the neurophysiological effects of synchronous TY and tDCS in stroke patients. Therefore, the application of cross-subject hemodynamic brain network methods to evaluate intervention-induced changes in network topology and functional integration may yield more comprehensive insights into the neural basis of rehabilitation.

Functional near-infrared spectroscopy (fNIRS) is a non-invasive optical imaging technique that quantitatively assesses the intensity of neural activity by real-time monitoring changes in the concentrations of oxygenated hemoglobin (HbO) and deoxygenated hemoglobin (HbR) in the cerebral cortex (19). With its high motion tolerance and excellent temporal resolution, fNIRS has become an ideal tool for dynamically evaluating cortical activation patterns during interventions (20). Therefore, the present study employed fNIRS technology to explore and compare differences in cortical responses and brain network in left-sided stroke patients with hemiplegia under three intervention modes: TY alone, tDCS alone, and synchronous TY + tDCS. The aim of this study was to reveal the network reorganization characteristics and specific neural mechanisms of different interventions, thereby generating objective, quantifiable, multi-level neuroimaging evidence for optimizing post-stroke upper limb rehabilitation programmes.

## 2 Materials and methods

### 2.1 Participants

From September to December 2024, 13 patients with post-stroke hemiplegia were recruited through screening of inpatients from the Xiamen Fifth Hospital.

#### 2.1.1 Inclusion criteria

(1) Confirmed stroke lesions by cranial CT or MRI examination. (2) First onset of stroke, or previous lacunar infarction without sequelae. (3) Left hemispheric stroke with right-sided hemiplegia. (4) Aged 18–80 years. (5) Disease duration of 1 month to 3 years with stable condition. (6) Right-handedness confirmed by the Edinburgh Handedness Inventory (EHI). (7) Upper limb Brunnstrom stage ≥II, hand function ≥I. (8) Ability to complete seated TY training with the affected hand supporting a 46 cm diameter yoga ball with the assistance of the unaffected hand. (9) Possession of static sitting balance ability, and ability to understand test instructions and cooperate with relevant tests.

#### 2.1.2 Exclusion criteria

(1) History of traumatic brain injury or central nervous system diseases (e.g., epilepsy, Alzheimer's disease, brain tumors). (2) Implanted metal or electronic devices in the body. (3) Skull defect. (4) Cardiac pacemaker implantation. (5) Severe cognitive impairment. (6) Mental or emotional disorders preventing cooperation with examinations. (7) History of skin or scalp injury or allergies.

#### 2.1.3 Withdrawal and dropout criteria

(1) Occurrence of significant discomfort during intervention, preventing continuation of the study. (2) Voluntary withdrawal from the study by the participant. (3) Failure to complete all interventions and related tests. (4) Poor quality of fNIRS data, rendering it unsuitable for subsequent analysis.

All participants provided informed consent and underwent screening according to predefined inclusion and exclusion criteria. This study was approved by the Ethics Committee of Xiamen Fifth Hospital (Approval No. XMWY-KY-2024-046) and conducted in strict accordance with the ethical standards specified in the 1975 Declaration of Helsinki (revised in 2008). The study has been registered with Chinese Clinical Trial Registry (Registration No. ChiCTR2400088853).

### 2.2 Intervention protocols

**Figure 1:**
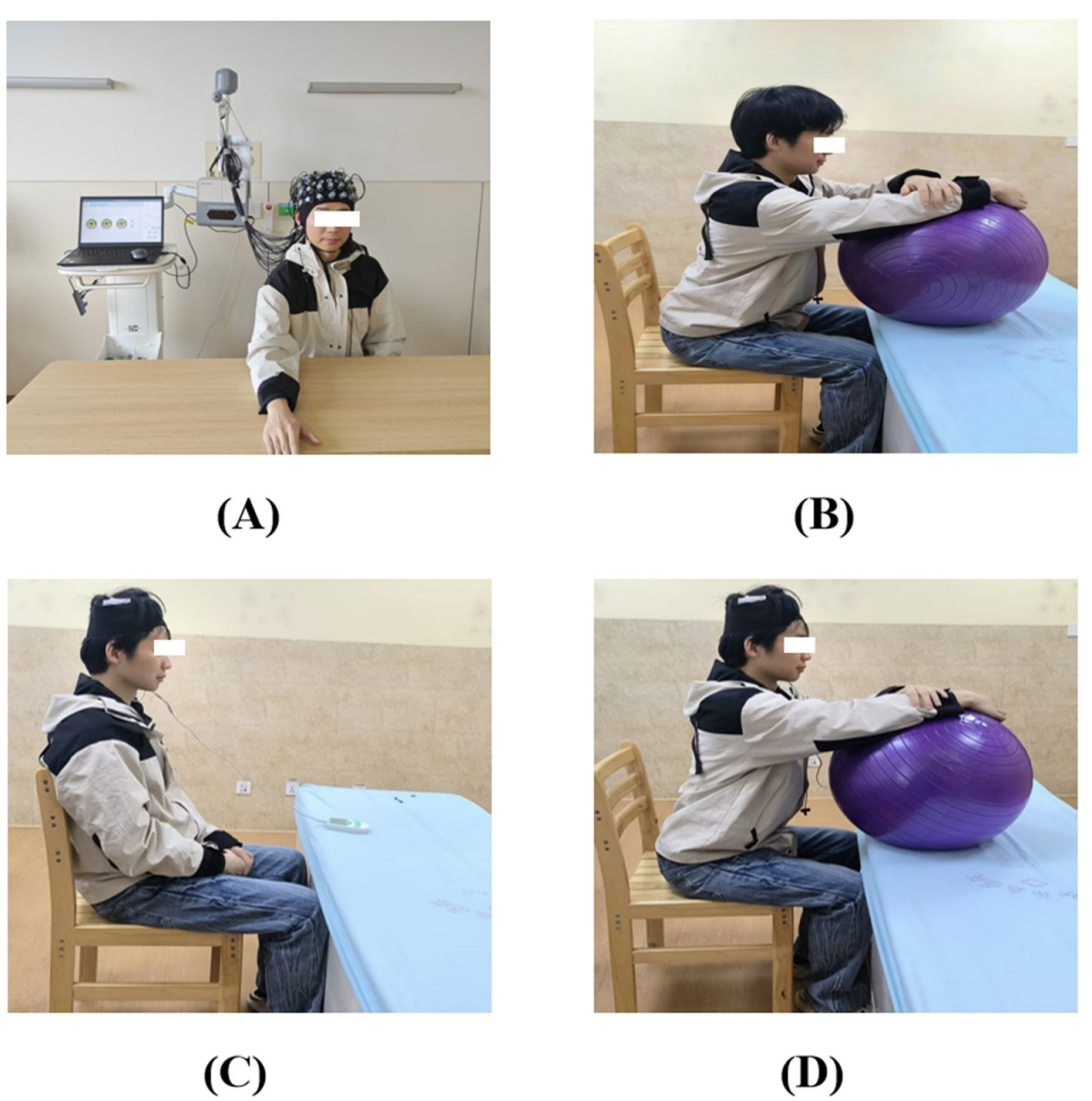
Experimental setup and three intervention scenarios. **(A)** Experimental setup. **(B)** TY alone. **(C)** tDCS alone. **(D)** Synchronous TY + tDCS. The member of the research team as the demonstrator.

Participants completed three brief intervention protocols in random order (Figure 1B, 1C, 1D): (1) TY alone: 20 min of standardized TY practice; (2) tDCS alone: 20 min of tDCS stimulation; (3) synchronous TY + tDCS: simultaneous tDCS stimulation and TY practice for 20 min. A 2-day washout period was set between each intervention.

#### 2.2.1 TY

Participants adopted a standard seated position with hips and knees flexed at 90°. The affected arm was naturally extended, with the forearm resting on a 46 cm diameter yoga ball. The therapist or the participant used the unaffected hand to assist in extending the palm of the affected hand and placing it stably downward onto the ball surface. The palm of the unaffected hand was then gently placed over the dorsum of the affected hand, with both hands supporting the yoga ball together. Guided by the unaffected hand, the affected upper limb was assisted to push the yoga ball to complete the TY movement. The movement required the upper limb to perform three-dimensional clockwise arc movements relying on the yoga ball, with smooth and continuous motion, accompanied by 15°-30° of trunk axial rotation and natural left-to-right transfer of the ischial sitting center of gravity. Movement frequency was uniformly controlled at approximately 0.5 Hz using a metronome. Participants maintained natural breathing throughout the training to ensure standardized movements and coordinated rhythm.

#### 2.2.2 tDCS

A professional device from Wuhan Yimai Medical Technology Co., Ltd. (China) was used, targeting the bilateral M1 regions. 4 × 4 cm saline-soaked sponge electrodes were applied, with the anode positioned over the left M1 region and the cathode symmetrically placed over the right M1 region. Stimulation parameters were set as follows: intensity of 2.0 mA for 20 min (21).

### 2.3 fNIRS measurement

**Figure 2:**
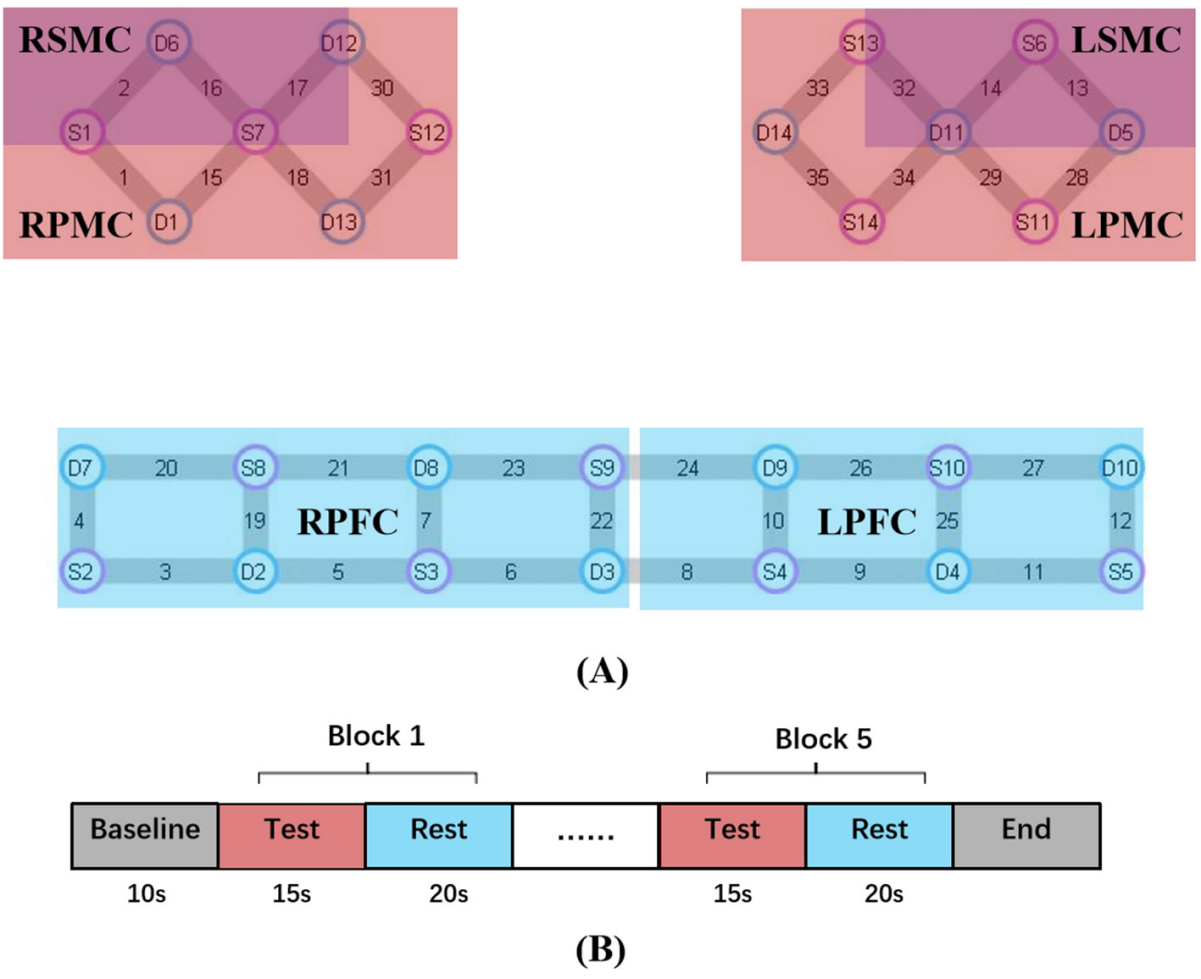
Correspondence between each channel and region of interest (ROI) and schematic illustration of the step-by-step detection process for HbO dynamic characteristics of each channel based on fNIRS. **(A)** fNIRS Channels and ROIs. The channels divided into the prefrontal cortex (PFC), premotor and supplementary motor cortex (PMC), and sensorimotor cortex (SMC). R/L, right/left. **(B)** fNIRS experimental paradigm.

Participants underwent fNIRS testing before and after each intervention. A NirSmart-6000A system (Danyang Huichuang Medical Equipment Co., Ltd., China) was used for multi-channel fNIRS data collection. Optical parameters included dual-wavelength (730/850 nm) LED light sources and avalanche photodiode (APD) detectors. Spatial configuration consisted of a 35-channel matrix formed by 14 light sources × 14 detectors with an inter-probe distance of 3.0 ± 0.3 cm. Sampling parameter was an 11 Hz acquisition rate. This study primarily observed the dynamic characteristics of HbO in the left and right prefrontal cortex (LPFC, RPFC), premotor and supplementary motor cortex (LPMC, RPMC), and sensorimotor cortex (LSMC, RSMC). The concentration of HbO was reported in units of millimoles per liter (mmol/L). The mapping between the region of interest (ROI) and the fNIRS channel was illustrated in Figure 2A.

Participants sat leaning on a stable chair with hips and knees flexed, maintaining a relaxed state. With the assistance of the study's researchers, they wore a fiber optic cap and looked straight ahead (Figure 1A). Once prepared, participants rested quietly for 10 s. Upon hearing an auditory cue, the study's researchers assisted them in performing passive elbow flexion-extension movements at a frequency of approximately 1 Hz for 15 s. Subsequently, an auditory cue indicated a 20-s rest period, and this cycle was repeated five times (Figure 2B).

### 2.4 fNIRS data preprocessing

HbO was used as the indicator of hemodynamic response because it is more sensitive to changes in cortical activity than HbR (22). The fNIRS data were processed using NirSpark software (Danyang Huichuang Medical Equipment Co., Ltd.) (23, 24). Raw light intensity data were first converted to optical density change data, and signal segments of poor quality were discarded. Then, spline interpolation algorithm was used to detect and correct motion-induced artifacts (24). Band-pass filter parameters were adjusted to 0.01–0.2 Hz to remove physiological noise. Optical density data were then converted to HbO concentration changes according to the Beer-Lambert law. Baseline correction of the fNIRS data was performed using hmrBlockAvg function from NirSpark (25), and block-averaged HbO changes for each measurement channel across three brief interventions were calculated. Finally, the difference values between post-intervention and pre-intervention HbO concentrations (ΔHbO) in the same ROI for each intervention across all participants were used for subsequent data analysis.

### 2.5 Formal data analysis

Formal data analysis was conducted using R (version 4.5.1). The present study employed a two-scale analytical framework, partitioning the analysis into channel-level cortical activation and cross-subject-level hemodynamic brain networks, to evaluate intervention effects comprehensively.

### 2.6 Methods of channel-level cortical activation analysis and power analysis

To characterize the spatial distribution of intervention effects, the activation analysis was conducted at the channel-level. Each fNIRS channel was treated as a distinct spatial sampling unit, effectively treating the channel as a block in a generalized randomized block design. This approach preserved the subtle and local hemodynamic specificity that might be obscured by individual. For each region of interest (ROI), the Friedman test was performed to compare ΔHbO across the three brief interventions, followed by *post-hoc* pairwise comparisons using Nemenyi test that employed the studentized range distribution. Nemenyi test implemented a single-step procedure that inherently controlled the family-wise error rate (FWER) by accounting for the maximum standardized rank difference across all pairwise comparisons. Consequently, the resulting *p*-values were already multiplicity-adjusted. This channel-level strategy aligned with the voxel-wise analysis logic in other neuroimaging studies (e.g., functional magnetic resonance imaging studies), where spatial sampling units were analyzed to detect brain regional response (26). To complement the inferential testing, effect sizes and statistical power were further evaluated following Tomczak (27). Specifically, the Friedman test statistic was approximated using an F distribution, from which Cohen's f was derived to quantify effect size. Power analysis was subsequently conducted to determine: (i) the observed effect size f_obs_, (ii) the actual power corresponding to f_obs_, and (iii) the critical effect size f_crit_ required to achieve 80% power for each ROI.

### 2.7 Methods of cross-subject-level hemodynamic brain network analysis

To perform hemodynamic brain network analysis at the cross-subject level, average ΔHbO features were first extracted from ROIs of all included participants under the three brief interventions. These pooled features were then used to construct the cross-subject hemodynamic brain networks via the Graphical Least Absolute Shrinkage and Selection Operator (GLASSO) within the High-dimensional Undirected Graph Estimation (HUGE) framework (28). Functional community detection was performed using the spin glass method with the optimal number of clusters determined automatically. And brain network layout was visualized using Davidson-Harel method. Nodal centrality metrics, edge betweenness, and global indicators of the hemodynamic brain networks mentioned above were also analyzed.

**Figure 3:**
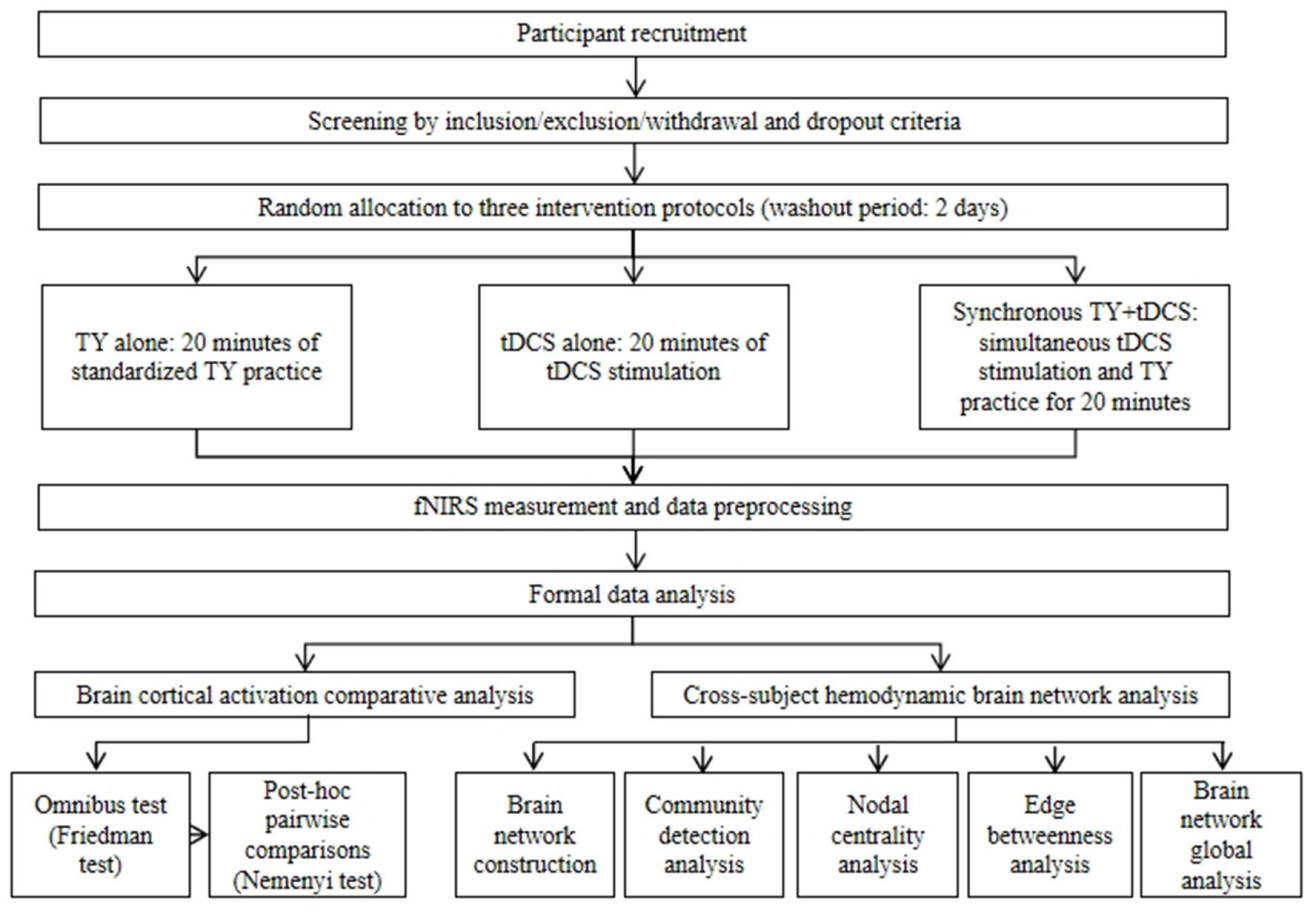
Experimental flow chart.

Research Flowchart shows in Figure 3.

## 3 Results

The characteristics of the participants are summarized in Table 1. Three subjects dropped out due to discharge before completing all interventions. A total of 10 subjects were finally included in the statistical analysis. No adverse events were reported among all participants during the entire study period.

**Table 1:** Baseline characteristics of participants.

Baseline variables	Value
Age (years)	56.00 [52.50,61.00]
Gender (Male/female)	7/3
Stroke type (I/H)	5/5
Post-stroke period (months)	14.00 [5.00,23.30]
Right upper limb (brunnstrom stage ranges)	II-V
Right hand (brunnstrom stage ranges)	II-V

Values described by the median (Q1, Q3), range or ratio. I, ischemic; H, hemorrhagic.

### 3.1 Channel-level cortical activation analysis

ΔHbO for each ROI under the three intervention modes was shown in Table 2. Significant differences in ΔHbO among interventions were found in the LPFC, RPFC, and LPMC, but not for RSMC, LSMC, and RPMC. Specifically, in LPFC and RPFC, the brief intervention of the synchronous TY + tDCS induced superior activation compared to tDCS alone. In LPMC, the brief interventions of tDCS alone and synchronous TY + tDCS both induced superior activation compared to TY alone.

**Table 2:** The activation level among different ROIs under the three intervention modes described by the median (Q1, Q3).

ROI	TY alone	tDCS alone	Synchronous TY + tDCS	*F*-value	*p*-value
LPFC	0.003 (−0.013, −0.022)	−0.008 (−0.040, −0.014)	0.006 (−0.015, 0.032)	5.805	< 0.01^c^
LSMC	−0.001 (−0.020, 0.015)	−0.004 (−0.016, 0.017)	0.010 (−0.013, 0.023)	1.455	0.242
LPMC	−0.008 (−0.037, 0.004)	0.003 (−0.017, 0.028)	0.003 (−0.010, 0.020)	10.961	< 0.001^a, b^
RPFC	0.004 (−0.013, 0.018)	−0.005 (−0.032, 0.024)	0.015 (−0.011, 0.034)	3.996	< 0.05^c^
RSMC	0.003 (−0.045, 0.011)	−0.001 (−0.024, 0.031)	−0.001 (−0.020, 0.011)	1.961	0.15
RPMC	−0.003 (−0.017, 0.007)	−0.004 (−0.028, 0.014)	0.007 (−0.017, 0.020)	2.212	0.115

Regarding the annotations of “a, b, c” in this table (the p-values already multiplicity-adjusted in Nemenyi test): “^a^”indicating the statistically significant difference between TY alone and tDCS alone (p < 0.05); “^b^”indicating the statistically significant difference between TY alone and the synchronous TY + tDCS (p < 0.05); “^c^”indicating the statistically significant difference between tDCS alone and the synchronous TY + tDCS (p < 0.05). ROI, region of interest; TY alone, 20 min of standardized TY practice; tDCS alone, 20 min of tDCS stimulation; Synchronous TY + tDCS, simultaneous tDCS stimulation and TY practice for 20 min; PFC, prefrontal cortex; PMC, premotor and supplementary motor cortex; SMC, sensorimotor cortex; R/L, right/left.

### 3.2 Power analysis

To verify the sufficiency of the sample size, power analysis was performed (Table 3). For the statistically significant ROIs, the channel-level analysis demonstrated relatively robust statistical power. In LPMC, the observed effect size (f_obs_ = 0.473) substantially exceeded the critical effect size for 80% power (f_crit_ = 0.315), yielding power of 0.99. In LPFC, the observed effect size (f_obs_ = 0.255) slightly exceeded the threshold (f_crit_ = 0.234), resulting in power of 0.87. In RPFC, the observed effect size (f_obs_ = 0.201) was marginally below the threshold (f_crit_ = 0.221), with power of 0.72.

**Table 3:** Power analysis results for detecting intervention effects on cortical activation in each ROI.

ROI	f_obs_	f_crit_	Power
LPFC	0.255	0.234	0.870
LSMC	0.224	0.412	0.308
LPMC	0.473	0.315	0.991
RPFC	0.201	0.221	0.716
RSMC	0.260	0.412	0.402
RPMC	0.212	0.316	0.450

For the statistically non-significant ROIs, the observed effect size (f_obs_ range: 0.212–0.260) were consistently smaller than the critical effect size for 80% power (f_crit_ range: 0.316–0.412). Consequently, the powers of these statistically non-significant ROIs were low (power range: 0.308–0.450). This suggested that the null findings in these regions were attributable to genuinely smaller intervention effects rather than insufficient channel numbers.

### 3.3 Cross-subject-level hemodynamic brain network analysis

#### 3.3.1 Connection structure of hemodynamic brain networks

**Figure 4:**
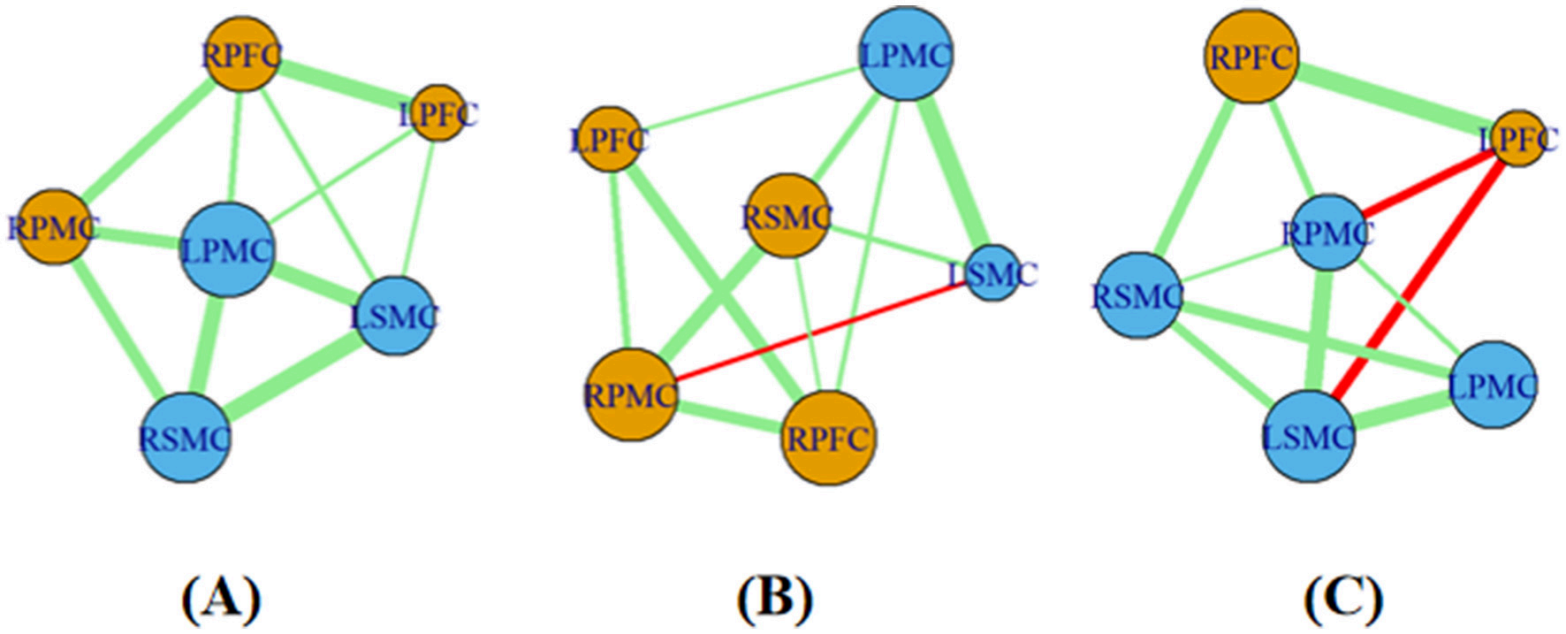
Hemodynamic brain network structures of three intervention modes. **(A)** Hemodynamic brain network graph of the brief intervention of TY alone. **(B)** Hemodynamic brain network graph of the brief intervention of tDCS alone. **(C)** Hemodynamic brain network graph of the brief intervention of the synchronous TY + tDCS. Each node representing a kind of ROI. Each edge representing functional connectivity between two ROIs. Node sizes corresponding to Strength values of ROI within the Hemodynamic brain networks (larger nodes indicating greater Strength) of the three intervention modes. For edge color, green edges representing positive connections, red edges representing negative connections. Edge thickness indicateing strength of connectivity between the two nodes (thicker edges representing stronger connectivity). For node color, nodes sharing the same color belonging to the same functional community.

The hemodynamic brain networks for all three intervention modes consisted of 6 nodes, 2 functional communities (the optimal number of clusters as 2) and 11 edges, as shown in Figure 4. Under the brief intervention of TY alone, all connections between ROIs were positive, with LPMC, LSMC, and RSMC forming one functional community and the remaining ROIs forming another. Under the brief intervention of tDCS alone, connections were positive except for the negative connection between LSMC and RPMC, with LPMC and LSMC forming one community and the remaining ROIs forming another. Under the brief intervention of the synchronous TY + tDCS, connections were positive except for negative connections between LPFC and RPMC, and between LPFC and LSMC, with LPFC and RPFC forming one functional community and the remaining ROIs forming another.

#### 3.3.2 Nodal centrality metrics

Centrality metrics (Closeness, Strength, PageRank) for each ROI under the three interventions were shown in Table 4. Under the brief intervention of TY alone, RPMC had the highest Closeness value (0.035), while LPMC had the highest Strength value (0.976) and PageRank value (0.213). Under the brief intervention of tDCS alone, RSMC and RPMC tied for the highest Closeness value (0.038), while RPMC had the highest Strength value (1.084) and PageRank value (0.194). Under the brief intervention of the synchronous TY + tDCS, LSMC had the highest Closeness value (0.049), Strength value (1.298) and PageRank value (0.221). The Closeness values for all ROIs under the brief intervention of the synchronous TY + tDCS were higher compared to the other two intervention modes.

**Table 4:** The node centrality in cross-subject hemodynamic brain networks under the three intervention modes specifically measured by 3 metrics: closeness, strength, and pagerank.

ROI	TY alone			tDCS alone			Synchronous TY + tDCS		
	Closeness	Strength	PageRank	Closeness	Strength	PageRank	Closeness	Strength	PageRank
LPFC	0.020	0.395	0.106	0.024	0.664	0.136	0.041	0.889	0.116
LSMC	0.025	0.731	0.163	0.023	0.761	0.140	0.049	1.298	0.221
LPMC	0.031	0.976	0.213	0.028	0.917	0.184	0.039	0.750	0.159
RPFC	0.027	0.679	0.167	0.032	0.931	0.183	0.037	0.888	0.200
RSMC	0.032	0.901	0.196	0.038	0.833	0.165	0.038	0.715	0.156
RPMC	0.035	0.670	0.154	0.038	1.084	0.194	0.038	0.869	0.148

ROI, region of interest; TY alone, 20 min of standardized TY practice; tDCS alone, 20 min of tDCS stimulation; Synchronous TY+tDCS, simultaneous tDCS stimulation and TY practice for 20 min; PFC, prefrontal cortex; PMC, premotor and supplementary motor cortex; SMC, sensorimotor cortex; R/L, right/left.

#### 3.3.3 Edge betweenness

The edge betweenness values for the three brief intervention modes were shown in Table 5. Under the brief intervention of TY alone, the connection LPFC-LPMC had the highest edge betweenness (edge betweenness = 4). Under the brief intervention of tDCS alone, the connection RPFC-RSMC had the highest edge betweenness (edge betweenness = 6). Under the brief intervention of the synchronous TY + tDCS, the connection RPMC-RSMC had the highest edge betweenness (edge betweenness = 7).

**Table 5:** The edge betweenness values of all of pairs of ROIs in cross-subject hemodynamic brain networks under the three intervention modes.

Intervention modes	Pairs of ROIs	Betweeness values
TY alone	LPFC–LPMC	4
	LPFC–LSMC	3
	LPFC–RPFC	0
	LPMC–LSMC	0
	LPMC–RPFC	2
	LPMC–RPMC	2
	LPMC–RSMC	3
	LSMC–RPFC	3
	LSMC–RSMC	1
	RPFC–RPMC	2
	RPMC–RSMC	1
tDCS alone	LPFC–LPMC	4
	LPFC–RPFC	0
	LPFC–RPMC	3
	LPMC–LSMC	0
	LPMC–RPFC	5
	LPMC–RSMC	0
	LSMC–RPMC	4
	LSMC–RSMC	5
	RPFC–RPMC	0
	RPFC–RSMC	6
	RPMC–RSMC	0
Synchronous TY + tDCS	LPFC–LSMC	1
	LPFC–RPFC	0
	LPFC–RPMC	4
	LPMC–LSMC	0
	LPMC–RPMC	5
	LPMC–RSMC	0
	LSMC–RPMC	0
	LSMC–RSMC	4
	RPFC–RPMC	5
	RPFC–RSMC	0
	RPMC–RSMC	7

ROI, region of interest; TY alone, 20 min of standardized TY practice; tDCS alone, 20 min of tDCS stimulation; Synchronous TY+tDCS, simultaneous tDCS stimulation and TY practice for 20 min; PFC, prefrontal cortex; PMC, premotor and supplementary motor cortex; SMC, sensorimotor cortex; R/L, right/left.

#### 3.3.4 Global network metrics

The three brief interventions differentially affected global network characteristics, as reflected by transitivity and modularity (Table 6). For transitivity, both TY alone and synchronous TY + tDCS exhibited a value of 0.677, which was higher than that of tDCS alone (0.600). For modularity, synchronous TY + tDCS showed the highest value (0.280), followed by tDCS alone (0.250), whereas TY alone showed the lowest value (0.180).

**Table 6:** The global network characteristics of cross-subject hemodynamic brain networks under the three intervention modes specifically measured by 2 metrics: transitivity and modularity.

Intervention modes	Transitivity	Modularity
TY alone	0.677	0.180
tDCS alone	0.600	0.250
Synchronous TY + tDCS	0.677	0.280

TY alone, 20 min of standardized TY practice; tDCS alone, 20 min of tDCS stimulation; synchronous TY+tDCS, simultaneous tDCS stimulation and TY practice for 20 min.

## 4 Discussion

This study employed channel-level and cross-subject-level brain science methodology by fNIRS to investigate the transient neurophysiological effects of TY, tDCS, and their synchronous application in left-hemispheric stroke patients with hemiplegia. By quantifying cortical activation and cross-subject hemodynamic brain network topology, we identified intervention-specific neuromodulatory patterns and, critically, elucidated a synergistic neuroplasticity mechanism induced by synchronous TY + tDCS.

### 4.1 Differential effects of the three brief interventions on cortical activation patterns

#### 4.1.1 Synergistic enhancement effect of synchronous TY + tDCS on LPFC and RPFC

The PFC is a core brain region for motor planning, attention allocation and, cognitive control, and its dysfunction directly affects motor learning and recovery after stroke (29–31). A key finding of this study is that the brief intervention of synchronous TY + tDCS exhibited significantly greater activation amplitude in the LPFC and RPFC compared with tDCS alone, a result attributed to the efficient coupling of tDCS-mediated neuromodulation, which lowers neuronal action potential thresholds, and TY-driven motor training, thereby enhancing cortical responsiveness (32). As a specific task requiring sustained attention, complex movement planning and multi-joint coordination, TY not only improved the neural microenvironment by reducing levels of inflammatory factors (TNF-α, IL-6) and alleviating oxidative stress (33, 34) but also promoted BDNF-mediated synaptic remodeling through behavioral driving (35). The simultaneous implementation of the two maximizes the mobilization of LPFC and RPFC resources, resulting in significantly superior activation effects compared with single neuromodulation.

#### 4.1.2 Dominant regulatory role of tDCS on LPMC

The LPMC is primarily responsible for motor preparation, programming and coordination, particularly playing a key role in organizing complex sequential movements of the upper limbs. Its damage often leads to delayed motor initiation and coordination disorders in stroke patients (36). The results of this study show that the brief interventions of tDCS alone and the synchronous TY + tDCS both exhibited significantly higher activation levels in the LPMC compared with TY alone, indicating that the neuromodulatory effect of tDCS was the main contributor to the transient activation of this brain region. The tDCS stimulation targeting the left M1 region could indirectly regulate the activation level of the ipsilateral LPMC through inter-cortical connections, consistent with the research conclusion by Li et al. (37) that tDCS combined with sensorimotor training could specifically activate the LPMC in stroke patients and was associated with improved upper limb function.

### 4.2 Synergistic characteristics of cross-subject hemodynamic brain networks

#### 4.2.1 Specific remodeling of cross-subject hemodynamic brain network under three intervention modes

While the cross-subject hemodynamic brain networks under all three intervention modes consisted of six core brain region nodes and two functional communities, the community partitioning and association patterns differed. TY alone formed a bipartite community structure: a “Motor Execution Community” (LPMC, LSMC, RSMC) and a “Cognitive Control Community” (RPFC, LPFC, RPMC), with all connections being positive. This suggests that TY training might promote positive synergistic interactions within the motor circuitry, providing a stable neural foundation for motor control. The continuous, coordinated limb movements in TY, integrating multisensory inputs, significantly strengthened functional connectivity between LPMC and LSMC, forming a tighter motor execution core community (38). Previous research showed that Tai Chi practice could enhance functional connectivity in specific brain regions (e.g., LPFC-RSMC), correlating with attention focus and movement perception (39). The intervention mode of tDCS alone consisted of LPMC and LSMC as the main community, with the remaining ROIs forming a secondary community. The brain network exhibited a negative correlation between LSMC and RPMC, possibly reflecting tDCS modulation of bilateral M1 excitability balance, inhibiting excessive compensation from the unaffected hemisphere to create conditions for functional reorganization in the affected motor cortex (40). Synchronous TY + tDCS exhibited a distinguished community partitioning pattern from TY alone. The key distinction lay in the incorporation of RPMC into the motor execution community, while the cognitive community comprised exclusively LPFC and RPFC. This segregation aligns with the established role of the dorsolateral prefrontal cortex (DLPFC) as an executive coordinator subserving higher-order cognitive functions including planning, working memory, and cognitive flexibility (41). The DLPFC regulates cognitive control via cortico-striato-thalamo-cortical circuits and often shows negative correlation with the default mode network (42, 43). In our study, this purely cognitive community composition in synchronous TY + tDCS might indicate that PFC could focus more on advanced cognitive functions while reducing direct involvement in motor execution, achieving more efficient segregation and synergy between cognitive control and motor execution systems compared to TY alone.

#### 4.2.2 Optimized improvement of brain region centrality under the synchronous TY + tDCS

Comparing the Closeness values of all ROIs in the intervention of synchronous TY + tDCS with those in the other intervention modes, it was found that the global accessibility of any brain region in the cross-subject hemodynamic brain network of this intervention mode was superior to the latter two on the whole. This optimization might reflect a synergistic mechanism whereby tDCS established a neuroplastic foundation, and Tai Chi provided high-intensity sensorimotor engagement, jointly facilitating the LSMC to emerge as more robust and functionally dominant network hub (44, 45).

#### 4.2.3 Functional significance of edge betweenness and global indicators

Edge between analysis in this study showed that the edge betweenness value of the RPMC-RSMC connection in synchronous TY + tDCS reached 7, which was not only dominant within this cross-subject hemodynamic brain network but also exceeded the highest record of any other single intervention mode. This topological characteristic further confirmed the unique advantage of the synchronous combined intervention in optimizing motor execution. Compared with TY alone, which was predicated solely on endogenous neuroplasticity, or tDCS alone, which was predicated solely on external electrical stimulation, synchronous TY + tDCS could significantly improve sensorimotor synergy efficiency.

In terms of global indicators for synchronous TY + tDCS, the network transitivity was above 0.60, indicating a relatively strong tendency toward triangular closure. The network modularity was approximately 0.30, indicating a certain degree of community structure within the cross-subject hemodynamic brain network, with a relatively balanced demand for functional differentiation and collaboration. These two global indicators together revealed that the cross-subject hemodynamic brain network under the intervention mode possessed dual characteristics of “small-world” local clustering and “dual-center” global organization. In this intervention mode, relatively high network transitivity might be attributed to the effect of tDCS (46), while the network modularity tendency might benefit from TY (47, 48).

Due to its highest Closeness value, the LSMC might serve as the hub node connecting different functional communities in the cross-subject hemodynamic brain network, with its topological position rather than connectivity strength dominating global efficiency. The synchronous combined intervention might enhance sensorimotor integration and neural remodeling effects through the network targeting role of LSMC in the whole network.

### 4.3 Limitations

To the best of our knowledge, this study might be the first to utilize fNIRS to explore the transient neural effects and cross-subject hemodynamic brain network profiling of comprehensive intervention of synchronous TY and tDCS. However, it still has some limitations. Firstly, the final sample size included in the analysis of this study was small, and all participants were from a single center, which limits the statistical power and external generalizability of the results. Secondly, this study only evaluated the transient cortical activation and the transient changes from cross-subject hemodynamic brain networks after different intervention modes, unable to track cumulative efficacy of these modes, nor directly correlating functional neuroimaging indicators with clinical improvements in upper limb motor function. Finally, the spatial resolution of fNIRS is subject to physical constraints, which may restrict the sensitive detection of activation in the deep primary SMC within brain sulci and long-distance connections of hemodynamic brain networks (49, 50). Future research should prioritize large-scale, multi-center trials integrating multimodal neuroimaging with prolonged longitudinal clinical follow-up to elucidate the sustained clinical gains and the mechanisms of dynamic trajectory of cross-subject hemodynamic brain network remodeling underlying this comprehensive intervention.

## 5 Conclusion

Using fNIRS, this study systematically revealed the neuromodulatory effects of TY alone, tDCS alone, and synchronous TY + tDCS from dual perspectives of channel-level cortical activation and cross-subject-level hemodynamic brain network analysis in patients with left-hemispheric stroke and hemiplegia. The results showed that in the aspect of cortical activation, synchronous TY + tDCS significantly enhanced cortical activation level in the LPFC and RPFC compared with tDCS alone, and exhibited comparable activation level in the LPMC to tDCS alone, both of which were higher than TY alone. In the aspect of cross-subject-level hemodynamic brain network analysis, synchronous TY + tDCS achieved efficient synergy between the cognitive regulation and motor execution networks by reshaping community structure, strengthening the core position of LSMC, improving network information transmission efficiency, and optimizing overall topological properties. This study provides objective neuroimaging evidence for optimizing post-stroke upper limb rehabilitation programmes and supports synchronous TY + tDCS as a potential and powerful option for future stroke rehabilitation.

## Data Availability

The original contributions presented in the study are included in the article/supplementary material, further inquiries can be directed to the corresponding authors.
